# “From an HCV and HIV point of view, it's been remarkable”: A qualitative study about using prescribed safer supply to support people who use drugs along the HIV and HCV prevention and treatment cascades in Ontario, Canada

**DOI:** 10.1002/jia2.70038

**Published:** 2025-10-08

**Authors:** Adrian Guta, Katherine Rudzinski, Marilou Gagnon, Rose A. Schmidt, Gillian Kolla, Danielle German, David Kryszajtys, Melissa Perri, Andrea Sereda, Christopher Sterling‐Murphy, Carol Strike

**Affiliations:** ^1^ School of Social Work University of Windsor Windsor Ontario Canada; ^2^ Dalla Lana School of Public Health University of Toronto Toronto Ontario Canada; ^3^ School of Nursing University of Victoria Victoria British Columbia Canada; ^4^ Division of Population Health and Applied Health Sciences Faculty of Medicine Memorial University St. John's Newfoundland and Labrador Canada; ^5^ Department of Health Behavior and Society Bloomberg School of Public Health Johns Hopkins University Baltimore Maryland USA; ^6^ London InterCommunity Health Centre London Ontario Canada

**Keywords:** addiction, HIV, HCV, harm reduction, opioids, people who use drugs (PWUD), safer supply

## Abstract

**Introduction:**

Despite advances in HIV and hepatitis C virus (HCV) treatment, people who use drugs (PWUD) face significant barriers along prevention and treatment cascades. Safer supply programmes (SSPs) providing prescribed pharmaceutical alternatives to the unregulated drug supply may create opportunities for enhanced healthcare engagement and person‐centred care.

**Methods:**

We conducted a qualitative study examining four SSPs in Ontario, Canada between February and October 2021. Semi‐structured interviews were conducted with 52 patients and 21 providers (including physicians, registered nurse practitioners, nurses and allied health professionals). Interviews explored experiences with safer supply and HIV/HCV care. Analysis used thematic techniques guided by the Consolidated Framework for Implementation Research.

**Results:**

SSPs supported HIV/HCV care by first addressing patients’ substance use needs, which created subsequent opportunities for building trust for broader health engagement. Providers identified the safer supply model as giving PWUD something they wanted, which then opened opportunities to discuss HIV, HCV, and other sexually transmitted and blood‐borne infections. SSPs provided opportunities to support patients with HIV and HCV testing and treatment initiation, and safer supply medications were bundled with HIV and HCV medications to support adherence. Non‐punitive approaches helped overcome previous negative healthcare experiences by prioritizing patient autonomy. Implementation challenges included balancing flexible, patient‐directed care with programme requirements and coordinating comprehensive services around individual needs.

**Conclusions:**

SSPs may improve HIV/HCV care delivery for PWUD by building services around their priorities and lived realities. The integration of safer supply with HIV/HCV care through daily dispensing and wraparound services showed promise for engaging people previously disconnected from care. While findings suggested improved treatment outcomes, limitations included data collection during COVID‐19, limited representation of some populations and a focus on opioid‐only programmes. Research examining long‐term outcomes and programme sustainability is needed as SSPs face growing scrutiny and closure in Canada.

## 1 INTRODUCTION

Despite advances in HIV prevention and treatment in North America, delivering person‐centred care that fully engages key populations remains challenging. In Canada, approximately 65,270 people were living with HIV in 2020, with an incidence rate of 4.7 per 100,000 population [1]. All‐cause HIV diagnoses rose 35.2% from 2022 to 2023; injection drug use accounted for 18.2% and combined male‐to‐male sexual contact plus injection, 4.5%. The United States reported approximately 1.1 million people living with HIV in 2022 [2]. A parallel epidemic is occurring for the hepatitis C virus (HCV), with an estimated 214,000 people living with chronic HCV in Canada [3] and 2.4 million in the United States [4]. Injection drug use remains a significant concern for HIV and HCV transmission [5, 6], with an estimated 3.6 million people who inject drugs in the United States [7] and an estimated 171,900 in Canada [8]. However, this represents a small subset of all people who use drugs (PWUD), whether by injection or other methods [9, 10, 11]. In response, Canadian jurisdictions have implemented a range of drug treatment and harm reduction interventions—such as opioid agonist treatment (OAT), supervised consumption sites (SCS), needle–syringe programmes and naloxone distribution to curb drug‐related harms; HIV, HCV, and sexually transmitted and blood‐borne infections (STBBIs) transmission; and overdose deaths [12, 13, 14, 15, 16]. The United States has adopted some of these approaches, to varying degrees across states, despite considerable opposition and regulatory barriers [15, 17]. However, drug use—especially injection—is considered a significant barrier to optimal HIV and HCV outcomes [18, 19, 20, 21, 22, 23, 24].

The HIV and HCV cascades span testing, linkage, ongoing care, treatment initiation, adherence, and ultimately viral suppression (HIV) or sustained virologic response (HCV) [25, 26, 27, 28]. PWUD face barriers at each step, including stigma in healthcare settings, competing priorities (e.g. engagement in survival economies to support their drug use) and limited access to comprehensive harm reduction services [29, 30]. These barriers can be partially addressed through the integration of HIV and HCV services with OAT [31, 32, 33, 34]. In the Canadian context, OAT refers primarily to methadone and buprenorphine‐naloxone, with slow‐release oral morphine second‐line treatment [35]. In the United States, these medications are collectively termed medications for opioid use disorder (MOUD) and include extended‐release naltrexone [36, 37]. Although OAT is considered a first‐line treatment for opioid use disorder, engagement and retention remain low [38, 39]. OAT patients often cite punitive cultures—surveillance, medication withholding, mandatory counselling, inconvenient hours—as key barriers [40]. Fentanyl's dominance in the unregulated drug supply has heightened overdose deaths (52,544 in Canada, 2016–2024 [41]; 110,037 in the United States, 2023 [42]) and stalled cascade progress for PWUD. The short half‐life of fentanyl may increase HIV and HCV transmission risk due to more frequent injection and equipment sharing [43, 44]. Due to the potency of fentanyl, OAT is harder to initiate, and retention in treatment has dropped significantly [39, 45, 46, 47]. Additionally, COVID‐19 deepened inequities, substance use [48, 49, 50] and housing instability [51, 52, 53], generating a “syndemic” effect in which overdose, HIV/HCV risk, and poverty reinforce each other and disrupt care engagement [54, 55].

In response to the impacts of the drug toxicity crisis and related syndemics, there have been calls across Canada [56] and the United States [57] for prescribed alternatives to the toxic drug supply. Building on this, Health Canada sanctioned “safer supply,” defined as “prescribed medications to people who use drugs, overseen by a health care practitioner, with the goal of preventing overdoses and saving lives…provided in a less clinical and more flexible way compared to other care options for substance use” [58]. Safer supply programmes (SSPs) are novel healthcare interventions that provide prescribed pharmaceutical alternatives to the unregulated drug supply, including opioids, stimulants and benzodiazepines [55]. Most SSPs have operated through community health centres and other primary care settings, where providers can offer “wraparound services” [59], including primary care, mental health support, harm reduction, with medication dispensed through community pharmacies [60, 61, 62, 63, 64]. However, SSPs have also been provided through standalone clinics, shelters, harm reduction and SCS, and one programme dispensed medication through a biometric machine [60, 65, 66]. Most SSP patients have previously tried OAT [67], but show higher retention on safer supply [68]. The first organized SSP started in 2016 [69], but some physicians were offering prescribed alternatives on a case‐by‐case basis to support people living with HIV/HCV to remain in care during hospital admissions [70]. A quasi‐experimental study found that at baseline, 34.1% of patients entering SSPs were living with HIV (compared to 7.6% of the matched cohort), and 69.5% had an HCV diagnosis [71]. While SSPs do not require abstinence from injection or unregulated drug use, reductions in both have been reported [61, 72]. Most SSPs employ a person‐centred approach, simultaneously addressing substance use needs while facilitating access to essential health and social services, which supports improved health outcomes and reduces overdose and all‐cause mortality [61, 64, 73, 74]. Canada also has injectable OAT (e.g. injectable hydromorphone and diacetylmorphine), which may be considered a safer supply [59, 75], but these programmes have been slow to scale up due to the necessary infrastructure and costs [76].

Although most SSP studies note implications for HIV and HCV [56, 77], only one study has examined combining safer supply with HIV pre‐exposure prophylaxis (PrEP) [78]. In‐depth studies about the relationship between safer supply and the HIV and HCV care cascades are needed. Subsequently, we sought the perspectives of SSP staff and clients to understand the impact of the safer supply approach and structure on the HIV and HCV care continuums for PWUD.

## 2 METHODS

Between February and October 2021, we examined four Canadian SSPs (one in London, three in Toronto). These SSPs utilized a community‐based primary care model, where physicians and nurse practitioners prescribed “safer supply” in the form of daily observed doses of long‐acting opioids (e.g. slow‐release oral morphine) alongside unobserved take‐home doses of immediate‐release hydromorphone (Dilaudid), which could be self‐administered as needed (including via injection). Observed slow‐release dosing may reduces HIV/HCV risk by preventing repeated injections with the same equipment [79]. Medications were dispensed through community pharmacies, except one programme in our sample had a small “observed arm” for patients deemed high‐risk for overdose due to concurrent high alcohol or benzodiazepine use. These patients could use their medication as desired, but were asked to do so on site in an SCS connected to the SSP.

### 2.1 Data generation and analysis

We conducted semi‐structured interviews and questionnaires with SSP patients (we use the term “patient” because these are clinical programmes, but “client” was used by participants and appears in quotations) and providers. Interview guides were developed with input from community advisory members with lived experience and members of the larger team with clinical expertise, including safer supply prescribing. We then pilot‐tested the guides with one provider and two patients, incorporating their feedback. Providers were recruited via email and snowball sampling, starting with all prescribers, who then referred other staff. Patient recruitment was facilitated through flyers distributed by the research team during site visits or through providers within participating SSPs. The study adhered to standard research ethics protocols, with individual participants providing informed consent. The University of Toronto Research Ethics Board provided ethics approval (Protocol: 40140).

Provider questionnaires collected data on socio‐demographic and professional background. Interviews explored SSP implementation, patient enrolment, prescribing practices and HIV/HCV care. Patient questionnaires gathered socio‐demographic characteristics and substance use/medical history, while interviews examined SSP experiences and HIV/HCV outcomes. Audio‐recorded interviews (20–105 minutes) were transcribed verbatim. Patients and allied health staff received $40 honoraria; prescribers were not compensated.

Questionnaire data were analysed in SPSSv28; interviews underwent thematic analysis [80] in MAXQDA using codebooks based on the Consolidated Framework for Implementation Research constructs and emergent concepts. Each transcript was coded by one of three primary coders and reviewed by another for consistency. Team members (AG, KR, MG, CS) extracted HIV/HCV‐related data, met regularly to identify themes and select quotes, with broader team support for final analysis. Initial analysis concluded March 2022; HIV/HCV sub‐analysis ran December 2023–March 2024.

## 3 RESULTS

We conducted interviews with 52 SSP patients and 21 providers. Of these 52 patients, all experienced significant inequities: *n* = 31, 59.6% reported housing precarity (e.g. staying in shelters, hotels/motels or experiencing homelessness), 98.1% (*n* = 51) relied on social assistance or disability support programmes and 86.5% (*n* = 45) had a history of incarceration. While predominantly white (*n* = 41, 78.8%), we interviewed Indigenous (*n* = 9, 17.3%), Black (*n* = 1, 1.9%) and Latino (*n* = 1, 1.9%) patients. Participants identified as men (*n* = 29, 55.8%) and women (*n* = 23, 44.2%) with a mean age of 46.6 years (range 29–62).

All seven patient‐participants living with HIV (13.5%) were on antiretroviral therapy (ART) and had achieved viral suppression. Of the 40 patient‐participants (76.9%) ever‐diagnosed with HCV, 20 (50%) had completed treatment, while 19 (47.5%) had never received treatment. Forty‐five SSP participants (86.5%) had tried methadone, and 12 (23.1%) were currently receiving OAT alongside safer supply (i.e. hydromorphone to supplement their needs) (see Table 1).

**Table 1:** Patient socio‐demographic information

	*N* (52)	%
**Programme site**		
London InterCommunity Health Centre	21	40.4%
Parkdale Queen West Community Health Centre	15	28.8%
South Riverdale Community Health Centre	11	21.2%
Street Health	5	9.6%
**Gender identity** ^a^		
Man	29	55.8%
Woman	23	44.2%
**Indigenous** ^b^		
No	42	80.1%
Yes, First Nation	8	15.4%
Yes, Métis	2	3.8%
**Race** ^b^		
White	41	78.8%
Indigenous	9	17.3%
Black	1	1.9%
Latino	1	1.9%
**Age**		
Mean	46.60	
Std. deviation	9.59	Range (29–62)
**Housing (past year)**		
An apartment, house or condo (Rented)	18	34.6%
Staying with friends, family or partner	13	25.0%
Shelters	7	13.5%
A hotel/motel or room rented by the night/week or month	6	11.5%
Homeless^c^	3	5.8%
An apartment, house or condo (Owned)	2	3.8%
Supportive or transitional group housing	2	3.8%
In a long‐term care facility	1	1.9%
**Income source (past year)** ^d^		
Social support (ODSP/OW)	51	98.1%
Paid job	9	17.3%
Other illegal activities	6	11.5%
Other government programme	5	9.6%
Sex work	3	5.8%
**Jail or prison**		
Ever	45	86.5%
Past year	3	5.8%
**HIV**		
Positive diagnosis	7	13.5%
Currently on medication^e^	7	13.5%
Undetectable viral load	7	13.5%
**HCV**		
Positive diagnosis	40	76.9%
**HCV medication** ^f^		
Yes—finished the treatment	20	38.5%
No—never	19	36.5%
Yes—now	1	1.9%
**Previous engagement with addiction treatment**		
Methadone	45	86.5%
Detox	17	32.7%
Live in	28	53.8%
Buprenorphine	22	42.3%
Outpatient	17	32.7%
Treatment in jail	3	5.8%
**Engagement with OAT** ^g^		
No—not currently	36	69.2%
Yes—currently	12	23.1%
No—never	2	3.8%

Abbreviations: ART, antiretroviral therapy; HCV, hepatitis C virus; HIV, human immunodeficiency virus; OAT, opioid agonist treatment; ODSP, Ontario Disability Support Program; OW, Ontario Works (provincial social assistance programme).

^a^ All patients reported being cisgender.

^b^ Race/Ethnicity was gathered using a two‐part question based on guidance provided by the Ontario Government. Participants were first asked if they identify as Indigenous (First Nations, Inuit or Métis), and second, what race category best describes them. Note: A person who identified as Indigenous could self‐report they were a different race category (e.g. white), and participants who did not identify as part of an Indigenous group in Canada could select Indigenous for their race (e.g. from an Indigenous group in another country).

^c^ Homelessness was defined as “living on the street, abandoned building, tent/encampment, car/vehicle, outside.”

^d^ Participants could select all that applied.

^e^ Among patients living with HIV.

^f^ Among patients ever diagnosed with HCV.

^g^ Data missing from two participants.

The 21 provider participants interviewed represented different roles within multidisciplinary care teams, including prescribers (i.e. four physicians (19.0%), five nurse practitioners (23.8%)), five registered nurses (23.8%) and seven allied health professionals (33.3%) (e.g. community health workers, case managers). Most providers had at least 6 years of experience working with PWUD (16 providers) and people living with HIV (15 providers) (see Table 2).

**Table 2:** Provider demographics and practice history

	*N* (21)	%
**Profession**		
Nurse practitioner	5	23.8%
Registered nurse	5	23.8%
Physician	4	19.0%
Community health worker	2	9.5%
Client care support, SSP administrator	1	4.8%
Health/systems navigator	2	9.5%
Outreach worker/site coordinator	1	4.8%
SSP case manager	1	4.8%
**Site**		
Parkdale Queen West Community Health Centre	9	42.9%
London InterCommunity Health Centre	6	28.6%
South Riverdale Community Health Centre	3	14.3%
Street Health	3	14.3%
**Gender identity**		
Woman	13	61.9%
Man	6	28.6%
Nonbinary	2	9.5%
**Indigenous** ^a^		
No	42	80.1%
**Race** ^a^		
White	15	71.4%
East Asian	2	9.5%
Indigenous Hispanic	1	4.8%
Mixed	1	4.8%
Southeast Asian	1	4.8%
Black	1	4.8%
**Age (category)** ^b^		
25–29	1	4.8%
30–34	7	33.3%
35–39	5	23.8%
40–44	3	14.3%
45–49	3	14.3%
55–59	1	4.8%
**Years worked—at site**		
Less than 6 months	1	4.8%
6–11 months	5	23.8%
1–2 years	3	14.3%
3–5 years	5	23.8%
6–10 years	6	28.6%
More than 10 years	1	4.8%
**Years worked—HIV**		
1–2 years	1	4.8%
3–5 years	4	19.0%
5–10 years	1	4.8%
6–10 years	9	42.9%
More than 10 years	6	28.6%
**Years worked—PWUD**		
1–2 years	1	4.8%
3–5 years	3	14.3%
5–10 years	1	4.8%
6–10 years	10	47.6%
More than 10 years	6	28.6%
**Hours worked**		
20–34	2	9.5%
35–39	10	47.6%
40–44	5	23.8%
45–49	2	9.5%
More than 55	2	9.5%
**Number of continuing education courses on substance use or harm reduction**		
0	3	14.3%
1	3	14.3%
2	6	28.6%
3	7	33.3%
5	2	9.5%

Abbreviations: HIV, human immunodeficiency virus; PWUD, people who use drugs; SSP, safer supply programme (clinical model under study).

^a^ Race/Ethnicity was gathered using a two‐part question based on guidance provided by the Ontario Government. Participants were first asked if they identify as Indigenous (First Nations, Inuit or Métis), and second, what race category best describes them. Note: A person who identified as Indigenous could self‐report they were a different race category (e.g. white), and participants who did not identify as part of an Indigenous group in Canada could select Indigenous for their race (e.g. from an Indigenous group in another country).

^b^ Note that one practitioner did not provide their age.

In our presentation of results, we first describe the patient sample's structural vulnerability and medical complexity, followed by the programme model, safer supply as an engagement mechanism, the HIV and HCV cascades, person‐centred care within the model, and implementation considerations and challenges. We noted during analysis that while all participants were at risk of HIV/HCV (e.g. past and ongoing injection drug use), responses to those questions were generally very short. We have focused on a subset of participants who were comfortable discussing their HIV/HCV care.

### 3.1 Structural vulnerability, medical complexity and HIV/HCV risk

Participating SSPs served a demographically diverse patient population characterized by complex medical and psychosocial needs with elevated HIV and HCV risk profiles. Healthcare providers consistently identified the profound medical complexity of their patient population:

“Many risks of death, either from intravenous drug use or complex other medical concerns. Extreme mental health concerns that we can tie in their safe supply medication with HIV meds [and] hepatitis. We try to look at the whole picture and say who's at most risk right now.” (Allied Health, 1)

A notable challenge was the widespread lack of engagement in healthcare services, with many patients avoiding medical care for extended periods. One patient's response exemplified this disengagement:

“I'd never really had other doctors. I've never been a person that goes – I wait till I'm almost dead before I get any help.” (Patient 1, Woman, age: 48)

Initial clinical assessments frequently revealed previously undiagnosed or untreated conditions. One nurse described their SSP's intake process:

“Some people have started on the program who have unmanaged medical conditions, so there's some clients who have HIV who have not been on medication for a long time… When we do baseline bloodwork, we do some of the hepatitis serology, so we'll know if people need Hepatitis A or Hepatitis B immunization.” (Registered Nurse, 1)

The severity of untreated conditions was particularly evident among patients living with HIV. As one physician noted:

“We have had quite a few referrals from the hospital‐based HIV team, of people who were taking no therapy or—we've probably had at least five from the hospital team that had CD4s of zero and were imminently going to die.” (Physician, 1)

### 3.2 Safer supply model as engagement mechanism

To address patients’ complex needs, participating SSPs integrated HIV and HCV care into their delivery model. This started with the intake process, prioritizing individuals at elevated risk for HIV and HCV transmission:

“We have a priority system that includes persons who are pregnant… People who are engaged in survival sex work and are at high risk of harm due to that. And people who have had multiple overdose events. Also, people who have untreated HIV or AIDS go to the top of the priority list.” (Nurse Practitioner, 1)

These prioritization criteria were further refined to account for multiple intersecting vulnerability factors:

“So female, [Black, Indigenous, People of Colour], homeless or transiently housed or living alone. Or significant medical complications related to IV drug use. Things like uncontrolled HIV, Hep C” (Physician, 2).

SSPs served as an HIV and HCV cascade engagement mechanism by first responding to participants’ self‐identified drug use needs:

“When I say ‘engagement tool’, what I mean by that is we're giving patients something that's actually useful and wanted in their life… And when they experience us supporting them on their own terms of their own identified needs, that brings them back into health care in other ways.” (Physician, 1)

Providers recognized that obtaining opioids and preventing withdrawal were their patients’ primary concerns and addressed these needs first. However, subsequent visits for titration (adjusting the dose of opioids to an individualized, therapeutic dose sufficient to suppress withdrawal, manage pain and meet patient‐identified goals) and monitoring facilitated opportunities to build trust and engage patients in discussions about HIV and HCV. The SSP model (care and opioid medication) kept patients engaged in care:

“From a HepC and HIV point of view, it's been remarkable. I am someone who has done primary care for HIV, and was doing it before safer supply, and it was the really challenging part of my job… and doing the programme and being able to twin the medications alongside their long‐acting [opioid] that they're going to the pharmacy for every day has made providing HIV primary care so much easier.” (Physician, 2)

### 3.3 Integration of HIV and HCV care across the treatment cascades

Building on this engagement foundation, the programmes integrated HIV and HCV care across the prevention and treatment cascades. This integration created opportunities for testing, treatment initiation and ongoing care, as discussed below.

#### 3.3.1 Testing and early diagnosis

After establishing initial relationships with SSP patients, providers systematically incorporated HIV, HCV and other STBBI testing into care. Providers emphasized such comprehensive intake screening:

“[We] connect with the nurses who usually do the bloodwork. We'll get a history around [HCV] as well as HIV testing, how long ago they had been tested, if they know their status or not, and then get consent to test.” (Physician, 2)

“Even giving a baseline bloodwork just to see what's going on for people. Because we've had people that have popped up as HIV positive and we've had people that have popped up as HepC positive.” (Allied Health, 2)

Testing became a regular component of ongoing care:

“We diagnose quite a few people with Hep C and other STIs like syphilis and gonorrhea and then just general screen bloodwork. So a lot of people haven't had that done in a really long time, and they're accessing it. And we offer it every six months or so.” (Nurse Practitioner, 2)

Providers noted that SSPs helped patients overcome traditional barriers to testing:

“People are getting connected to more. I think people are doing things that they didn't normally do because of the stigma and the medical violence that they experience. So definitely cancer screenings and HIV testing and HepC testing and access to treatment and medication has all increased.” (Allied Health, 3)

#### 3.3.2 Treatment initiation and supporting adherence

Following diagnosis or re‐engagement in care, the programmes provided structured support for treatment initiation through the integrated service model:

“I've seen a number of people get connected… we do labwork to get those results, people know their status, and then it's really up to them if they want to go forward with treatment, definitely increases acceptability to all those options… Then, once they can stabilize on their [safer supply] dose, it makes it much more likely that ART will be successful.” (Registered Nurse, 2)

Prescribers could also bundle HIV/HCV medications with daily dispensing of opioid medications, accounting for varying levels of support:

“Also starting people on Hepatitis C treatment, and we had a few people who had never been taking any treatment for HIV, and with this, it was facilitated. And just the fact that they have to go to the pharmacy every day anyways. So, picking up those extra pills was no big deal, and they were willing to do it.” (Registered Nurse, 3)

For participants requiring additional support, programmes worked with pharmacies to coordinate medication dispensing:

“For those folks that aren't great at taking their meds, we will tend to do blister packs with the pharmacies, so that, in order for them to get their safer supply meds, they have to either – they get their HIV or HepC meds or psychiatric meds.” (Allied Health, 1)

Participants confirmed the integrated approaches’ benefit. When asked if the SSP influenced their decision to start HCV treatment, one participant explained:

“For me it's been positive keepin’ me – my health, I know where I'm at. I am going to be doing my Hep C for the new year. So that's something I'm going to be doing. My next appointment with the doctor, I'll be do my blood and that, find out where my liver is at. They [care team] just told me to let them know when I'm ready for it, and then we'll do it.” (Patient 2, Woman, age: 45)

While patients could choose their pharmacies, some of the SSPs had relationships with pharmacies that were familiar with the patient population and the types of scripts (especially high medication dosages), which expanded the care team and reduced medication disruptions.

### 3.4 Engagement and retention in care

SSPs established a foundation for sustained HIV/HCV care engagement by addressing clinical needs and structural barriers. Providers described using the SSP to help stabilize patients and facilitate ongoing care coordination:

“So, HIV has a couple of prongs, we have an in‐house HIV testing [programme]. So [the programme] would send them to us if the patients aren't able to take their therapy because of the chaos going into their street drug use, so we'll stabilize them with safe supply and observe the HIV treatment, and then we concurrently manage them with [the program] and the infectious‐disease doctor.” (Physician, 1)

This integrated approach significantly reduced treatment interruptions, with this physician contrasting the stability of SSP patients compared to previous challenges with patients needing to restart treatment multiple times:

“Whereas previously, people were coming in, would need to be restarted, have genotype testing done over and over again, deal with infection complications, and that is just not happening. And then when it comes to HepC, almost everyone ends up knowing what their status is, if people are positive [we] line them up to repeat that bloodwork in six months, or find a previous test that was positive so they can get started on treatment.” (Physician, 2)

Patients emphasized how the daily opioid dispensing model supported their HIV/HCV medication adherence:

Interviewer: When you go to the pharmacy to get your Dilaudid, do you also pick up HIV medication at the same time?

Participant: At the same time, yes, thanks god yes, thanks god.

Interviewer: How do you feel about that?

Participant: Great, can you imagine? That's my life. I would not be able to – I don't think – I'd be dead, put it that way, if it wasn't for [safer supply], yes. (Patient 3, Male, age: 53)

Another patient explained how the programme model (with daily opioid medication pick‐up and wraparound services), in combination with HIV medication bundling, provided a routine he could follow to remain engaged:

“I thought I was going to have problems with my routine of going to the drugstore. I love going to take my HIV meds every day when I do that. So now I have my doctor put on – when I go for anything, ‘Don't give him his drugs unless he gets his HIV medication’. You know what I mean? It's great… I was overwhelmed by all the support and help everybody's given me over the years. I feel – I tell people I don't deserve it, but I've gotten better at that. Yeah, I do deserve it.” (Patient 4, Male, age: 53)

However, providers noted that not all patients required this level of support:

“I think they were fairly adherent to begin with, these folks, actually. I think that it could potentially make a difference if they're going to the pharmacy every day and even – could they pick up their HIV meds with the safer supply meds. But the folks that we have had in who are HIV positive were already connected to a provider and were doing all right.” (Nurse Practitioner, 2)

#### 3.4.1 Improved treatment outcomes

The comprehensive care model contributed to improvements in HIV and HCV treatment outcomes. One nurse practitioner discussed clinical data demonstrating the programme's impact:

“Out of all patients who came to safer supply with a palliative designation, a hundred per cent of them are still alive and no longer palliative. The patients who came to us with untreated HIV, that had actually progressed to AIDS, ninety‐six per cent were virally suppressed, and on continuous HIV treatment. Out of all patients who came to us with Hepatitis C diagnoses, 92%… had either received Hepatitis C treatment or were in the process of receiving Hepatitis C treatment.” (Nurse Practitioner, 1)

These programme‐level outcomes were reflected in patient experiences. For example, one patient detailed their HIV viral suppression:

“I wasn't even takin’ my meds before I started the program and now, I'm undetectable and I'm over two hundred [CD4]. So it's good.” (Patient 5, Woman, age: 48)

This coordinated approach to HIV and HCV care, anchored by daily safer supply dispensing, created multiple opportunities for health system engagement while reducing traditional barriers to care. The integration of services supported patients across the whole cascade of HIV/HCV care, while maintaining flexibility to accommodate varying levels of need. The clinical outcomes suggest that SSPs effectively support the most medically complex patients in achieving and maintaining viral suppression and completing HCV treatment.

### 3.5 Person‐centred care approach to service delivery

The SSPs in our study implemented person‐centred approaches to care and service delivery that acknowledged how past healthcare experiences (including HIV and drug use stigma) influence current engagement in care. This approach was characterized by non‐judgemental, accessible care delivery, recognizing the complex needs and histories of those accessing these programmes. SSP patients emphasized how this approach differed markedly from their previous healthcare experiences. One patient highlighted the more personalized nature of care:

“You get that one‐on‐one…it's way more interactive, it's way more personal… it's way more humane than the other ones. It's just more judgmental‐free, you lay all your problems on the table. You know? I don't think the other ones even care to know your problems like that.” (Patient 6, Male, age: 29)

The co‐location and integration of services, combined with supportive relationships with providers, facilitated sustained engagement in care:

“My HIV doctor is here, so I can – everything's all in one place, counselling, HIV, right? And I can talk about the changes in my life, or my needs, and the staff… they're almost like family, you know what I mean? ‘Cause I see them so much’. And I really don't feel like I'm a second‐class citizen to anybody. And they treat people how they [want to be] treated, and the people that work here will go to the ends of the earth for you.” (Patient 7, Woman, age: 44).

Another patient described how this approach fostered ongoing engagement:

“I feel like one of the family here. They took care of my – I get teary when I think about it. They took care of my HepC. They called me, they made sure I'd come in, and they asked – I started going to the appointments downstairs, and then I started volunteering a little. It's just one thing leads to another to another, I feel like this is my family.” (Patient 8, Male, age: 49)

Overall, this patient‐centred approach was described by all participants as important for engaging and retaining PWUD in HIV/HCV care.

### 3.6 Implementation considerations and challenges

Integrating safer supply with HIV and HCV care raised several important implementation considerations. One significant concern involved ensuring that programme eligibility criteria did not inadvertently create perverse incentives around HIV and HCV treatment. With respect to prioritizing unmanaged HIV and HCV at intake, one physician explained:

“We're very careful not to put these criteria out there, so that clients don't end up purposely going off their treatments, for instance, for HIV and Hep C. That has been a reality at another program.” (Physician, 2)

Another key consideration involved the timing and coordination of various health and social services. Providers emphasized that SSPs must be integrated within a comprehensive care approach that simultaneously addressed multiple determinants of health, as one physician articulated:

“And what we know is that, if we don't do things concurrently, no one thing works well. Safe supply, at least the way we do in a community, is only partly about the drugs. But we can only take people so far, even with safe supply, if we're not addressing housing, food security, safety, unmanaged health conditions.” (Physician, 1)

Providers emphasized that successful integration of HIV and HCV services into SSPs required attention to not just safer supply and HIV/HCV medications, but also patients’ basic needs like housing to ensure programmes achieved their intended outcomes.

## 4 DISCUSSION

The SSPs we studied reached structurally vulnerable individuals, enabling HIV/HCV care for people typically disconnected from healthcare. In keeping with the growing body of literature about SSPs, those in our sample provided person‐centred care for PWUD and created an environment where PWUD felt valued and were supported in addressing their health needs [59, 60, 66, 68, 69, 71, 73, 75, 78, 81]. Our analysis contributes to this discussion by examining the relationship between SSPs and HIV/HCV. Although designed to prevent overdose by replacing the toxic supply, SSPs can also curb injection‐related HIV/HCV risk and foster person‐centred care [82]. While PWUD experience disproportionately high rates of HIV/HCV, they have been underserved by conventional care models [19, 29, 30, 83]. The HIV and HCV care cascade literatures focus on how to support PWUD to adapt and meet standard programme requirements and established clinical markers (e.g. viral load) [18, 19, 20, 21, 22, 23, 24]. In contrast, SSPs tailor service delivery around patients’ daily routines and priorities (including meeting their substance use needs and preventing withdrawal) [81], facilitating care engagement and entry points along the HIV and HCV cascades [84].

Our results highlight how effective HIV/HCV care delivery requires extensive relationship‐building before conventional cascade metrics become relevant [85]. Providers described the need to establish trust [64], and give patients something they want. This pre‐engagement phase—largely absent in traditional cascade frameworks—was crucial for achieving meaningful outcomes. The patient‐centred, relational and non‐punitive approach taken by providers proved particularly important for helping clients overcome histories of stigma and negative healthcare experiences and stay engaged in care, as has been called for in the literature [86, 87]. Our findings align with earlier work calling for recognizing patient goals and non‐linear cascade pathways, with multiple entry points reflecting people's diverse priorities and circumstances [88, 89, 90]. Safer supply participation improves OAT retention [91], and sustained OAT in turn enhances HIV and HCV cascade outcomes [92].

As noted, few patient participants discussed their HIV/HCV risk and care in detail. Despite considerable progress in anti‐stigma campaigns for HIV and HCV, especially the “U = U” movement [93], this messaging may not have reached marginalized PWUD. We posit that internalized stigma—combined with the immediate threat of fatal overdose in the current drug‐toxicity crisis—diminishes participants’ focus on the more abstract risks of HIV or HCV. For participants living with HIV, SSPs supported both initial diagnosis and re‐engagement in care, achieving impressive viral suppression rates among previously disengaged patients. Similarly, SSPs reported success for HCV in treatment initiation and completion. The integration of safer supply with HIV/HCV care offers several advantages aligned with person‐centred principles: addressing both immediate needs and longer‐term health goals; providing daily structure allowing for treatment initiation and adherence; and combining harm reduction with opportunities for healthcare engagement [94], which may support progress on nested HIV and HCV care continuums [95]. Recognizing the growing interest in prescribed safer supply in the United States [96], it is worth noting that while testing and diagnosis among PWUD are progressing, barriers remain to ART engagement and viral suppression [97]. Strategies to improve ART engagement are cost‐effective for reducing incidence [98], and low‐threshold access is critical for ending the HIV epidemic in the United States [99]. Maintaining low HIV incidence requires sustained ART engagement support, which is more cost‐efficient than large‐scale prevention programmes [100]. For HCV, innovative strategies [101] and comprehensive community‐based care are needed [102], while the United States may benefit from SSPs and other international approaches that address multiple barriers to achieving elimination [103].

Despite the documented success of SSPs across Canada [60], the model remains controversial, with efforts across the country to restrict and prevent their use [104, 105]. The lack of sustained funding threatens to reverse gains for many patients. Harm reduction service disruptions may lead to increased overdose risk, loss of trusted healthcare relationships and deterioration in social stability [106, 107, 108]. While critics have cited concerns about medication diversion [105], this can be effectively managed through non‐punitive approaches that maintain therapeutic relationships [109, 110, 111]. Debates surrounding SSPs (where fears about PWUD are used to justify restricting access to lifesaving medications) underscore the need to bridge the gap between evidence and policy in addressing both the overdose crisis and the ongoing HIV/HCV epidemics. Whereas HIV treatment‐as‐prevention and PrEP seemed radical when first introduced—reorienting how medications are used and to what ends—they are now part of comprehensive responses [112]. Radical models are needed to address the ongoing drug toxicity crisis, as slow iteration is not working [113]. The HIV sector has a long history of innovation and adaptation, working closely with those most affected to generate community‐relevant interventions that can be scaled across diverse contexts—safer supply is another HIV‐prevention tool in a growing toolbox.

### 4.1 Limitations

Our study has several methodological limitations that warrant consideration. Data collection occurred during COVID‐19, when SSPs faced unique operational challenges that may have influenced service delivery and participant experiences. We also cannot speak to longer‐term outcomes or programme sustainability. Additionally, despite programmes prioritizing diverse populations, our sample had limited representation of Black, Indigenous and trans individuals. The programmes we examined focused exclusively on opioid safer supply and did not offer prescribed stimulants. The SSP model discussed in this paper is one across a spectrum that includes community‐led compassion clubs [114, 115], which should also be considered for their overdose and HIV/HCV prevention potential. Finally, our study focused on SSPs in a single Canadian province [116], which may limit generalizability to other Canadian jurisdictions and other countries with different health and drug policy environments.

## 5 CONCLUSIONS

Our findings demonstrate how person‐centred SSPs can transform HIV/HCV prevention and treatment for structurally vulnerable individuals by building services around patients’ self‐identified priorities, preferences and lived realities. In this model, drug use‐related needs serve as the starting point and mechanism for engagement. The person‐centred approach used by SSP providers prioritized trust‐building and responsive care over rigid protocols, which enabled providers to maintain strong therapeutic relationships even when patient engagement fluctuated. Lessons may be transferable to other contexts dealing with the intersection of HIV and HCV, an increasingly toxic drug supply, and threats to harm reduction services (e.g. the United States, the UK, Australia). Future population‐level research is needed to examine the impact of safer supply along each stage of the HIV and HCV cascades, long‐term outcomes, compare drug options in trial conditions, consider the role of chronic pain [117], as well as optimal models for stimulant safer supply, and approaches to programme scale‐up that maintain fidelity to person‐centred principles. Most urgently, research is needed to document the health impacts of SSP closures, including HIV and HCV outcomes, to inform evidence‐based policy decisions about the model. There is a clear need to preserve and expand—rather than dismantle—novel services that have effectively reached populations historically underserved by mainstream healthcare models.

## COMPETING INTERESTS

The authors declare that they have no conflict of interest regarding the contents of this paper.

## AUTHORS' CONTRIBUTIONS

Dr. Guta and Dr. Strike had full access to all of the study's data and are responsible for its integrity and accuracy. For the larger study from which data for this paper are used, AG, CS, RAS, DK and MP collected the qualitative data and iteratively coded them. RAS conducted a statistical analysis. AG, KR and MG conceptualized this paper, with AG taking the lead on writing duties. GK, AS, DG and CS supported subsequent analysis, interpretation and writing. All authors meaningfully contributed to the writing of the paper and reviewed drafts.

## FUNDING

The study was funded by the Ontario HIV Treatment Network, EFP‐1113. Dr. Guta is supported by the Canada Research Chairs program.

## Data Availability

The data that support the findings of this study are available on request from the corresponding author. The data are not publicly available due to privacy or ethical restrictions.
